# Health-related quality of life in childhood arthritis: the impact of generic versus disease-specific instruments

**DOI:** 10.1186/s12969-026-01274-9

**Published:** 2026-09-12

**Authors:** Jelleke B. de Jonge, Sytze de Roock, Nele Grundhoefer, Deborah A. Marshall, Sebastiaan J. Vastert, Rae S. M. Yeung, Susanne M. Benseler, Joost F. Swart

**Affiliations:** 1 Department of Paediatric Rheumatology, Division of Paediatrics, Wilhelmina Children’s Hospital, Faculty of Medicine, University Medical Centre Utrecht, Utrecht University, Utrecht, The Netherlands; 2 Centre for Translational Immunology, University Medical Centre Utrecht, Utrecht University, Utrecht, The Netherlands; 3 Departments of Community Health Services and Medicine, McCaig Institute for Bone and Joint Health, Cumming School of Medicine, O’Brien Institute for Public Health, University of Calgary, Calgary, AB Canada; 4 Cumming School of Medicine, Alberta Children’s Hospital Research Institute, University of Calgary, Calgary, AB Canada; 5 Division of Rheumatology, Department of Paediatrics, Immunology and Institute of Medical Science, The Hospital for Sick Children, University of Toronto, Toronto, ON Canada; 6 Children’s Health Ireland, University College Dublin, Trinity College Dublin, Dublin, Ireland

**Keywords:** Children, Health-related quality of life, Arthritis, JIA, EQ-5D, JAMAR

## Abstract

**Background:**

Health-related quality of life (HRQoL) data provided by children with arthritis and their parents is critically important to enable co-design of care and shared decision making. However, there is significant heterogeneity in how HRQoL is captured, as both generic (EQ-5D-Y-5L) and disease-specific instruments (JAMAR) are available. This study aimed to examine the concordance of the JAMAR and EQ-5D-Y-5L questionnaires in childhood arthritis.

**Methods:**

The international UCAN CAN-DU study prospectively enrolled consecutive children with arthritis across Canada and the Netherlands. This study included patients in whom corresponding JAMAR and EQ-5D-Y-5L questionnaires were completed on the same date by either the patients or their parents. The concordance of the questionnaire responses was measured by comparing the answers given to five specific JAMAR domains corresponding to the EQ-5D-Y-5L dimensions (anxiety/depression; mobility; pain/discomfort; self-care; usual activities). The impact of disease activity was assessed. Spearman’s correlation explored the association between global scales from the JAMAR and the EQ-5D-Y-5L.

**Results:**

The study included 678 children with JIA; 63% were female; the median age at first questionnaire was 11.9 years (interquartile range: 6.2–15.1). Concordances between JAMAR and EQ-5D-Y-5L responses were found in 84% of the questions across all domains (84%, 82%, 85%, 87%, and 82% for anxiety/depression, mobility, pain/discomfort, self-care, and usual activities, respectively). In a state of inactive disease, generic and disease-specific instruments converged across all domains except for pain/discomfort. Comparison of global assessment scales revealed a high correlation between the EQ-5D-Y-5L and the JAMAR (Spearman’s correlation: *R* = -0.72, *p* < 0.001).

**Conclusions:**

Patients and parents provided different responses to generic and disease-specific HRQoL instruments. The concordance was reduced in all domains except for pain/discomfort when children were experiencing high disease activity, highlighting the perceived conceptual differences and limitations of HRQoL instruments.

**Supplementary Information:**

The online version contains supplementary material available at https://doi.org/10.1186/s12969-026-01274-9.

## Background

Juvenile Idiopathic Arthritis (JIA) is the most common chronic rheumatic disease in children [1]. The treatment of active joint inflammation in JIA has significantly improved over the past decades thanks to the availability of effective biologic therapies, in particular when these are initiated early [2, 3]. However, some children living with arthritis continue to experience pain, fatigue, impaired functioning, and difficulties participating in social life [4, 5]. JIA patients report significantly reduced health-related quality of life (HRQoL) not only compared to healthy children, but also those affected by other chronic illnesses [5, 6]. Measuring and addressing HRQoL is a critical component of care to capture both the individual and societal burden of childhood arthritis and to enable shared decision making.

The Juvenile Arthritis Multidimensional Assessment Report (JAMAR) is a validated disease-specific questionnaire to measure patient-reported outcomes designed for the comprehensive evaluation of children with JIA [7]. It effectively discriminates between healthy individuals and JIA patients across multiple populations [8]. The JAMAR includes components that assess health-related quality of life, along with broader information, including disease activity, severity, and medication usage in JIA patients [9]. The most commonly used generic instrument assessing HRQoL across diseases and populations is the EuroQol Five-Dimension questionnaire (EQ-5D) [10]. It explores and quantifies five dimensions: anxiety/depression, mobility, pain/discomfort, self-care, and usual activities [11]. The parent-proxy of the EQ-5D youth version with five response levels (EQ-5D-Y-5L) has recently been validated in children with JIA [12]. Additionally, the EQ-5D-Y-5L was found to have good diagnostic performance in children with JIA, with the ability to identify patients who potentially require treatment adjustments [13].

Both generic (EQ-5D) and disease-specific instruments (JAMAR) assess health-related quality of life in children with JIA. The relationship between these questionnaires is not yet fully understood. There is significant heterogeneity in how HRQoL is captured across the EQ-5D and the JAMAR, raising the question of whether these questionnaires provide complementary or redundant information. A better understanding of the relationship between generic and disease-specific questionnaires can help determine when both questionnaires are needed and minimize the assessment burden of patients by enabling a more targeted and informed selection of the instruments. Therefore, this study aimed to examine the concordance of JAMAR and EQ-5D-Y-5L questionnaires in childhood arthritis.

## Methods

The international multicentre UCAN CAN-DU study prospectively enrolled children with arthritis across Canada and the Netherlands. Patients with JIA were included in this study from the UCAN CAN-DU cohort if JAMAR and EQ-5D-Y-5L questionnaires were completed on the same calendar date by either the patients or their parents between May 2020 and February 2026. Physician- and patient/family-derived data were prospectively captured in the designated UCAN eHealth platform. The study was approved by the Institutional Research Ethical Board of the University Medical Centre Utrecht (no. 18–474) for the Netherlands and by the Conjoint Health Research Ethics Board, University of Calgary for Canada (REB17-1563).

### Demographics and clinical data

Demographic characteristics included age at symptom onset, biological sex, and country of residence. Treating physicians reported International League of Associations for Rheumatology (ILAR) JIA subtypes. Clinical variables included the overall active joint count (AJC). A joint was considered active if it was swollen or had both signs of tenderness and a limited range of motion. Active joints were assessed in 83 joints, as described previously [3]. The Physician Global Assessment (PhGA) of disease activity and Patient/Parent Global Assessment (PPGA) of well-being were captured using a Visual Analogue Scale (VAS) ranging from 0 to 10 points. Clinical Juvenile Arthritis Disease Activity Scores 10 joints (cJADAS10) were calculated based on the PhGA, PPGA, and AJC maximized to 10 active joints.

### Questionnaires

The EQ-5D-Y-5L is a generic HRQoL questionnaire for children aged 8–15 years, measuring the health status of ‘today’ through five dimensions (Anxiety/Depression, Mobility, Pain/Discomfort, Self-care, and Usual activities) [14]. A validated parent-proxy version of the EQ-5D-Y-5L is available for children with JIA [12]. EQ-5D-Y-5L dimensions are rated on a 5-point ordinal scale with ‘severity’ response options of no problems, a little bit of problems, some problems, a lot of problems, and extreme problems/cannot do [14]. In addition to the 5 dimensions, the EQ-5D-Y-5L asks, “We would like to know how good or bad your health is today”. The patient’s health is captured using a vertical VAS ranging from 0 (worst health you can imagine) to 100 (best health you can imagine) [15].

The JAMAR is a disease-specific questionnaire for children with JIA, measuring the health status with multiple domains [9]. The ‘Quality of life’ of the past four weeks is rated on a 4-point ordinal scale with ‘frequency’ response options of never, sometimes, often, and every day (and a “not-applicable” option, excluded from analysis in this study). Additionally, the following question is asked as part of the JAMAR: “Considering all the ways the illness affects you, please evaluate how you feel at the moment”. This question is answered by completing a horizontal VAS ranging from 0 (very well) to 10 (very poorly) [9].

In our study, patients and parents completed serial questionnaires between May 2020 and February 2026, with assessment intervals varying across participants. When both the patient and the parent completed the same questionnaire on the same date, the patient’s responses were used for the analysis. Questions completed by two parents, and not by the patient, were excluded from the analysis if their responses were discrepant. Observations with “not-applicable” responses in JAMAR were excluded from all analyses.

### Statistical analysis

Measurements of AJC, PhGA, PPGA and cJADAS10 taken within 14 days of questionnaires completion were included in the analysis. The value closest to the date of questionnaires completion was used in the analysis when multiple measurements for AJC, PhGA, PPGA or cJADAS10 were available within the 14-day interval. In this study, the five dimensions from the EQ-5D-Y-5L were compared with five JAMAR domains that most closely aligned in content (Supplementary Table 1).

First, the observed ordinal relationship between the EQ-5D-Y 5-point scale and the JAMAR 4-point scale, separately for each category, was reported. The observed absolute counts and the column percentages, representing the proportion of each JAMAR answer corresponding to a given EQ-5D-Y-5L response, were reported. Next, the total percentage of concordance in each domain was calculated by comparing dichotomized answers, distinguishing between no problems and any level of problems. No problems were defined as the JAMAR “never” responses and the EQ-5D-Y-5L “no problems” responses, while problems included all other response categories (JAMAR: sometimes, often, every day; EQ-5D-Y-5L: a little bit, some, a lot, extreme). Cohen’s kappa was used to assess agreement while accounting for agreement expected to occur by chance. A kappa score of 0.00-0.20 was considered to indicate slight agreement, 0.21–0.40 fair agreement, 0.41–0.60 moderate agreement, 0.61–0.80 substantial agreement, and 0.81-1.00 almost perfect agreement. To account for the non-independence of observations within patients, cluster-level bootstrap resampling (2000 replicates) was performed at the patient level, and bootstrap confidence intervals were calculated.

Additionally, comparisons were conducted between patient-completed and parent-completed JAMAR and EQ-5D-Y-5L questionnaires to evaluate whether respondent type influences the concordance between the two questionnaires. Moreover, analyses were conducted separately by disease activity states, defined as active (AJC > 0) and inactive (AJC = 0) arthritis, and active (PhGA ≥ 1) and inactive (PhGA < 1) disease, to analyse whether disease activity affects the concordance between the two questionnaires. AJC and PhGA were selected as a reflection of disease activity instead of cJADAS10, as cJADAS10 was relatively often missing due to incomplete PPGA scores. Additionally, the cJADAS10 is partially based on the PPGA, which is itself a patient/parent-reported measure. As two patient/parent-reported questionnaires are being compared, the preference is not to base the disease activity state on patient/parent-reported outcomes as well.

Spearman’s correlation was used to explore the association between the EQ-5D-Y-5L VAS and the JAMAR VAS. The following criteria were used to interpret the strength of the correlation: negligible (*r* < 0.3), low (0.3 ≤ *r* < 0.5), moderate (0.5 ≤ *r* < 0.7), high (0.7 ≤ *r* < 0.9), and very high (*r* ≥ 0.9) correlation [16]. All analyses were performed in R version 4.4.1 software [17] using the package tidyverse (version 2.0.0) [18].

## Results

A total of 678 children with JIA were included in the study; 428 were female (63%). The median age at the first questionnaire was 11.9 years (interquartile range (IQR): 6.2–15.1). The majority (415 patients, 61%) were cared for in Dutch centres. The median active joint count at the first questionnaire of each patient was 2.0 (IQR: 0.0–4.0), the median physician global assessment was 2.0 (IQR: 0.0-3.5), and the median patient/parent global assessment was 2.8 (IQR: 0.5–5.1) (Table 1). Of all patients with a known JIA subtype at their first visit, 51 children (11%) had enthesitis-related arthritis; 199 children (43%) had oligoarticular JIA; 114 (25%) had RF-negative polyarticular JIA; 27 children (6%) had RF-positive polyarticular JIA; 22 (5%) children were diagnosed with psoriatic arthritis, 35 (8%) with systemic JIA, and 16 (3%) with undifferentiated JIA. JIA subtype was not yet defined at the first visit for 214 patients (32%). The variables AJC, PhGA, PPGA and cJADAS10 contained missing data, with data completeness of 75%, 75%, 72% and 71%, respectively.

### Questionnaires

The majority of patients (374, 55%) completed the questionnaires once; 174 (26%) submitted both instruments concurrently twice, and 77 (11%) three times; 53 patients (8%) completed them more than three times, with a maximum of 11 times (Supplementary Fig. 1). The total number of questionnaires included in the study was 1206. Of the JAMAR questionnaires, 239 (20%) were completed by the patients and 967 (80%) by the parents. Similarly, 244 (20%) of the EQ-5D-Y-5L were completed by patients, and 962 (80%) by parents. Five questions (0.1%) were excluded from the analysis as two parents completed the question, but their responses were discrepant. Additionally, 224 questions (3%) were excluded from the analysis as “non-applicable” had been answered in one of the pre-selected categories of the JAMAR.

**Table 1:** Demographics and disease-related characteristics of children with juvenile idiopathic arthritis with paired generic and disease-specific health-related quality of life measures

	Total cohort *N* = 678
Age at first questionnaire	11.9 (6.2, 15.1)
Biological sex (Female)	428 (63)
Country of residence (Netherlands)	415 (61)
Number of paired JAMAR and EQ-5D-Y-5L questionnaires	1206
Number of paired questionnaires completed	1 (1, 2)
Active Joint Count^a^	2.0 (0.0, 4.0)
Physician Global Assessment^a^	2.0 (0.0, 3.5)
Patient/Parent Global Assessment^b^	2.8 (0.5, 5.1)
cJADAS10^c^	7 (3, 12)
JIA subtype^d^	
Enthesitis-related arthritis	51 (11)
Oligoarticular JIA	199 (43)
Polyarticular JIA, RF-	114 (25)
Polyarticular JIA, RF+	27 (6)
Psoriatic arthritis	22 (5)
Systemic JIA	35 (8)
Undifferentiated JIA	16 (3)

Legend: Disease-related values were based on the first time respondents completed generic and disease-specific measures. Values are presented as median (interquartile range) or n (%). Abbreviations: cJADAS10; clinical Juvenile Arthritis Disease Activity Score 10 joints. EQ-5D-Y-5L; EuroQol 5-Dimension Youth 5-Level. JAMAR; Juvenile Arthritis Multidimensional Assessment Report. JIA; Juvenile Idiopathic Arthritis. ^a^Data complete for 75%. ^b^Data complete for 72%. ^c^Data complete for 71%. ^d^Data complete for 68% (JIA subtype yet unknown at first visit)

### Distribution of EQ-5D-Y-5L responses by JAMAR response category

Comparisons were made between the EQ-5D-Y-5L and JAMAR questionnaires using their ordinal scales, and absolute count and column percentages were calculated (Table 2). Across all domains, a high proportion of patients who reported having problems “sometimes in the past four weeks” in the JAMAR reported “no problems today” in the EQ-5D-Y-5L (20%, 37%, 24%, 38%, and 23% for, respectively, the domains anxiety/depression, mobility, pain/discomfort, self-care, and usual activities) (Table 2). Surprisingly, a few patients reported in the JAMAR having “everyday problems in the past four weeks” and “no problems today” in the EQ-5D-Y-5L. Additionally, a proportion of the patients who reported in the JAMAR to have “never problems in the past four weeks” reported “a little bit of problems today” or even “some problems”, “A lot of problems” or “Extreme problems” in the EQ-5D-Y-5L.

**Table 2:** Domain-specific responses of JIA patients and their parents to generic (EQ-5D-Y-5L) and disease-specific (JAMAR) instruments

Domain			JAMAR			
			Never	Sometimes	Often	Everyday
Anxiety/depression	**EQ-5D-Y-5L**	No problems	545 (85)	88 (20)	3 (4.2)	1 (3.0)
		A little bit	88 (14)	256 (58)	11 (15)	6 (18)
		Some	6 (0.9)	87 (20)	33 (46)	10 (30)
		A lot	0 (0)	8 (1.8)	22 (31)	15 (45)
		Extreme	0 (0)	0 (0)	3 (4.2)	1 (3.0)
Mobility		No problems	544 (89)	120 (37)	14 (11)	9 (7.5)
		A little bit	60 (9.9)	148 (46)	37 (28)	27 (23)
		Some	3 (0.5)	53 (16)	63 (48)	41 (34)
		A lot	2 (0.3)	4 (1.2)	16 (12)	37 (31)
		Extreme	0 (0)	0 (0)	1 (0.8)	6 (5.0)
Pain/discomfort		No problems	287 (83)	119 (24)	6 (4.0)	3 (1.4)
		A little bit	52 (15)	266 (54)	44 (29)	28 (13)
		Some	4 (1.2)	94 (19)	75 (50)	82 (38)
		A lot	0 (0)	8 (1.6)	25 (17)	85 (39)
		Extreme	1 (0.3)	3 (0.6)	1 (0.7)	18 (8.3)
Self-care		No problems	778 (95)	89 (38)	8 (22)	5 (14)
		A little bit	28 (3.4)	114 (48)	12 (33)	5 (14)
		Some	4 (0.5)	29 (12)	11 (31)	10 (28)
		A lot	0 (0)	2 (0.8)	5 (14)	12 (33)
		Extreme	8 (1.0)	3 (1.3)	0 (0)	4 (11)
Usual activities		No problems	501 (82)	75 (23)	7 (7.5)	3 (3.7)
		A little bit	95 (15)	155 (48)	25 (27)	10 (12)
		Some	17 (2.8)	69 (21)	35 (38)	22 (27)
		A lot	1 (0.2)	20 (6.2)	25 (27)	35 (43)
		Extreme	0 (0)	3 (0.9)	1 (1.1)	11 (14)

Legend: Values presented as n (% of column total within each domain). Abbreviations: EQ-5D-Y-5L; EuroQol 5-Dimension Youth 5-Level. JAMAR; Juvenile Arthritis Multidimensional Assessment Report. JIA; Juvenile Idiopathic Arthritis

### Concordance between the EQ-5D-Y-5L and JAMAR

The overall percentage of concordance between the responses of the JAMAR and EQ-5D-Y-5L, identifying whether a patient experienced any problems or no problems, was 84%. Domain-specific concordances were 84% (Bootstrap confidence Interval (BI): 82%-86%), 82% (BI: 80%-85%), 85% (BI: 82%-87%), 87% (BI: 85%-89%), and 82% (BI: 80%-85%) for the anxiety/depression, mobility, pain/discomfort, self-care, and usual activities domains, respectively (Table 3). The Cohen’s kappa values were 0.68 (BI: 0.64–0.72), 0.65 (BI: 0.60–0.69), 0.65 (BI: 0.59–0.70), 0.66 (BI: 0.61–0.71), and 0.64 (BI: 0.59–0.69), respectively. Across all domains except for anxiety/depression and usual activities, the JAMAR noted problems in more reports than the EQ-5D-Y-5L. When the analysis was restricted to patient-completed JAMAR and EQ-5D-Y-5L questionnaires only, the observed percentages of concordance were 85% (BI: 79%-89%), 80% (BI: 74%-86%), 85% (BI: 80%-89%), 89% (BI: 85%-93%), and 80% (BI: 75%-85%) for anxiety/depression, mobility, pain/discomfort, self-care, and usual activities, respectively (Table 4). The corresponding Cohen’s kappa values were 0.69 (BI: 0.59–0.78), 0.60 (BI: 0.48–0.72), 0.60 (BI: 0.48–0.71), 0.70 (BI: 0.58–0.81), and 0.61 (BI: 0.50–0.70). Similarly, the observed percentages of concordance were 84% (BI: 82%-87%), 83% (BI: 81%-86%), 84% (BI: 82%-87%), 87% (BI: 85%-89%), and 83% (BI: 80%-85%), respectively, when the analysis was restricted to parent-completed JAMAR and EQ-5D-Y-5L questionnaires (Table 4). The Cohen’s kappa values were 0.69 (BI: 0.64–0.73), 0.66 (BI: 0.62–0.71), 0.65 (BI: 0.59–0.71), 0.66 (BI: 0.60–0.71), and 0.65 (BI: 0.60–0.70), respectively.

**Table 3:** Domain-specific concordance in responses comparing generic (EQ-5D-Y-5L) and disease-specific (JAMAR) instruments

Domain	Number (%) of patient/parents reporting no problems versus problems on the JAMAR and EQ-5D-Y-5L				Concordance (BI)	Cohen’s kappa (BI)
			JAMAR			
			No problems	Problems		
Anxiety/depression	**EQ-5D-Y-5L**	No problems	545 (46)	92 (8)	84% (82%-86%)	0.68 (0.64–0.72)
		Problems	94 (8)	452 (38)		
Mobility		No problems	544 (46)	143 (12)	82% (80%-85%)	0.65 (0.60–0.69)
		Problems	65 (5)	433 (37)		
Pain/discomfort		No problems	287 (24)	128 (11)	85% (82%-87%)	0.65 (0.59–0.70)
		Problems	57 (5)	729 (61)		
Self-Care		No problems	778 (69)	102 (9)	87% (85%-89%)	0.66 (0.61–0.71)
		Problems	40 (4)	207 (18)		
Usual activities		No problems	501 (45)	85 (8)	82% (80%-85%)	0.64 (0.59–0.69)
		Problems	113 (10)	411 (37)		

Legend: The numbers of patient/parents reporting no problems versus problems on the JAMAR and EQ-5D-Y-5L are presented as n (% of total within each domain). “No problems” were defined as responses of never on the JAMAR and no problems on the EQ-5D-Y-5L. “Problems” included all other response categories (JAMAR: sometimes, often, every day; EQ-5D-Y-5L: a little bit, some, a lot, extreme). Observed concordance was calculated based on dichotomized JAMAR and EQ-5D-Y-5L responses and presented as a percentage. Cohen’s kappa was calculated to assess agreement while accounting for agreement expected by chance. Cluster-level bootstrap resampling (2000 replicates) was performed at the patient level, and bootstrap confidence intervals were reported to account for non-independence of observations within patients. Abbreviations: BI; Bootstrap confidence Interval. EQ-5D-Y-5L; EuroQol 5-Dimension Youth 5-Level. JAMAR; Juvenile Arthritis Multidimensional Assessment Report

**Table 4:** Domain-specific concordance in responses comparing generic (EQ-5D-Y-5L) and disease-specific (JAMAR) instruments by respondent type

Domain	Number (%) of patient/parents reporting no problems versus problems on the JAMAR and EQ-5D-Y-5L						Concordance (BI)		Cohen’s kappa (BI)	
			JAMAR				Patient-reported	Parent-reported	Patient-reported	Parent-reported
			Patient-reported		Parents-reported					
			No problems	Problems	No problems	Problems				
Anxiety/depression	**EQ-5D-Y-5L**	No problems	106 (45)	18 (8)	431 (46)	72 (8)	85%	84%	0.69	0.69
		Problems	18 (8)	92 (39)	74 (8)	357 (38)	(79%-89%)	(82%-87%)	(0.59-0.78)	(0.64-0.73)
Mobility		No problems	104 (44)	25 (11)	435 (46)	116 (12)	80%	83%	0.6	0.66
		Problems	21 (9)	84 (36)	41 (4)	344 (37)	(74%-86%)	(81%-86%)	(0.48-0.72)	(0.62-0.71)
Pain/discomfort		No problems	42 (18)	30 (13)	240 (25)	97 (10)	85%	84%	0.6	0.65
		Problems	6 (3)	156 (67)	51 (5)	564 (59)	(80%-89%)	(82%-87%)	(0.48-0.71)	(0.59-0.71)
Self-Care		No problems	167 (71)	23 (10)	600 (68)	78 (9)	89%	87%	0.7	0.66
		Problems	2 (1)	42 (18)	36 (4)	164 (19)	(85%-93%)	(85%-89%)	(0.58-0.81)	(0.60-0.71)
Usual activities		No problems	91 (39)	19 (8)	405 (47)	64 (7)	80%	83%	0.61	0.65
		Problems	27 (12)	97 (41)	83 (10)	310 (36)	(75%-85%)	(80%-85%)	(0.50-0.70)	(0.60-0.70)

Legend: The numbers of patient/parents reporting no problems versus problems on the JAMAR and EQ-5D-Y-5L, by respondents, are presented as n (% of total within each domain). “No problems” were defined as responses of never on the JAMAR and no problems on the EQ-5D-Y-5L. “Problems” included all other response categories (JAMAR: sometimes, often, every day; EQ-5D-Y-5L: a little bit, some, a lot, extreme). Observed concordance was calculated based on dichotomized JAMAR and EQ-5D-Y-5L responses and presented as a percentage. Cohen’s kappa was calculated to assess agreement while accounting for agreement expected by chance. Cluster-level bootstrap resampling (2000 replicates) was performed at the patient level, and bootstrap confidence intervals were reported to account for non-independence of observations within patients. Abbreviations: BI; Bootstrap confidence Interval. EQ-5D-Y-5L; EuroQol 5-Dimension Youth 5-Level. JAMAR; Juvenile Arthritis Multidimensional Assessment Report

### Concordance between the EQ-5D-Y-5L and JAMAR per disease activity status

Among questionnaires for which the active joint count was known within a 14-day window around questionnaire completion (71%), concordance across the different arthritis states was assessed. The percentage of patients with no problems, according to both the JAMAR and EQ-5D-Y-5L questionnaires, was higher across all domains in patients with inactive arthritis than in those with active arthritis (Table 5). Additionally, more concordance between the EQ-5D-Y-5L and JAMAR questionnaires was found in JIA patients with inactive arthritis compared to those with active arthritis in all domains, except for pain/discomfort (Table 5). Similar results were found when comparing inactive disease (PhGA < 1) and active disease (PhGA ≥ 1) (Supplementary Table 2). In JIA patients with inactive arthritis, concordance percentages of 85% (BI: 82%-89%), 83% (BI: 79%-87%), 81% (BI: 76%-85%), 90% (BI: 87%-94%), and 84% (BI: 80%-88%) were found, respectively, for the domains of anxiety/depression, mobility, pain/discomfort, self-care, and usual activities. In JIA patients with active arthritis, concordance percentages of 83% (BI: 80%-87%), 81% (BI: 77%-84%), 86% (BI: 83%-89%), 83% (BI: 79%-86%), and 81% (BI: 77%-85%) were found. When patients with highly active arthritis (≥ 5 active joints) were compared with those with moderately active arthritis (1 to 4 active joints), concordance remained unchanged in the self-care domain but increased across all other domains.

**Table 5:** Domain-specific concordance in responses comparing generic (EQ-5D-Y-5L) and disease-specific (JAMAR) instruments by arthritis activity

Domain	Number (%) of patient/parents reporting no problems versus problems on the JAMAR and EQ-5D-Y-5L						Concordance (BI)		Cohen’s kappa (BI)	
			JAMAR				Inactive arthritis	Active arthritis	Inactive arthritis	Active arthritis
			Inactive arthritis		Active arthritis					
			No problems	Problems	No problems	Problems				
Anxiety/depression	**EQ-5D-Y-5L**	No problems	200 (57)	30 (9)	174 (35)	38 (8)	85%	83%	0.68	0.66
		Problems	21 (6)	97 (28)	46 (9)	241 (48)	(82%-89%)	(80%-87%)	(0.59-0.76)	(0.59-0.72)
Mobility		No problems	230 (66)	41 (12)	137 (27)	66 (13)	83%	81%	0.56	0.59
		Problems	18 (5)	61 (17)	31 (6)	265 (53)	(79%-87%)	(77%-84%)	(0.46-0.66)	(0.51-0.66)
Pain/discomfort		No problems	152 (43)	41 (12)	44 (9)	48 (9)	81%	86%	0.62	0.47
		Problems	26 (7)	132 (38)	23 (5)	391 (77)	(76%-85%)	(83%-89%)	(0.53-0.71)	(0.36-0.57)
Self-Care		No problems	285 (83)	30 (9)	258 (56)	54 (12)	90%	83%	0.54	0.62
		Problems	3 (1)	24 (7)	26 (6)	123 (27)	(87%-94%)	(79%-86%)	(0.39-0.69)	(0.54-0.69)
Usual activities		No problems	212 (63)	28 (8)	125 (27)	35 (8)	84%	81%	0.61	0.59
		Problems	25 (7)	70 (21)	52 (11)	248 (54)	(80%-88%)	(77%-85%)	(0.52-0.71)	(0.51-0.67)

Legend: The numbers of patients/parents reporting no problems versus problems on the JAMAR and EQ-5D-Y-5L, by arthritis state (inactive arthritis: AJC = 0; active arthritis: AJC > 0), are presented as n (% of total within each domain). “No problems” were defined as responses of never on the JAMAR and no problems on the EQ-5D-Y-5L. “Problems” included all other response categories (JAMAR: sometimes, often, every day; EQ-5D-Y-5L: a little bit, some, a lot, extreme). Observed concordance by arthritis state was calculated based on dichotomized JAMAR and EQ-5D-Y-5L responses and presented as a percentage. Cohen’s kappa was calculated to assess agreement while accounting for agreement expected by chance. Cluster-level bootstrap resampling (2000 replicates) was performed at the patient level, and bootstrap confidence intervals were reported to account for non-independence of observations within patients. Abbreviations: BI; Bootstrap confidence Interval. EQ-5D-Y-5L; EuroQol 5-Dimension Youth 5-Level. JAMAR; Juvenile Arthritis Multidimensional Assessment Report

### High correlation between generic and disease-specific global assessments

**Fig. 1:**
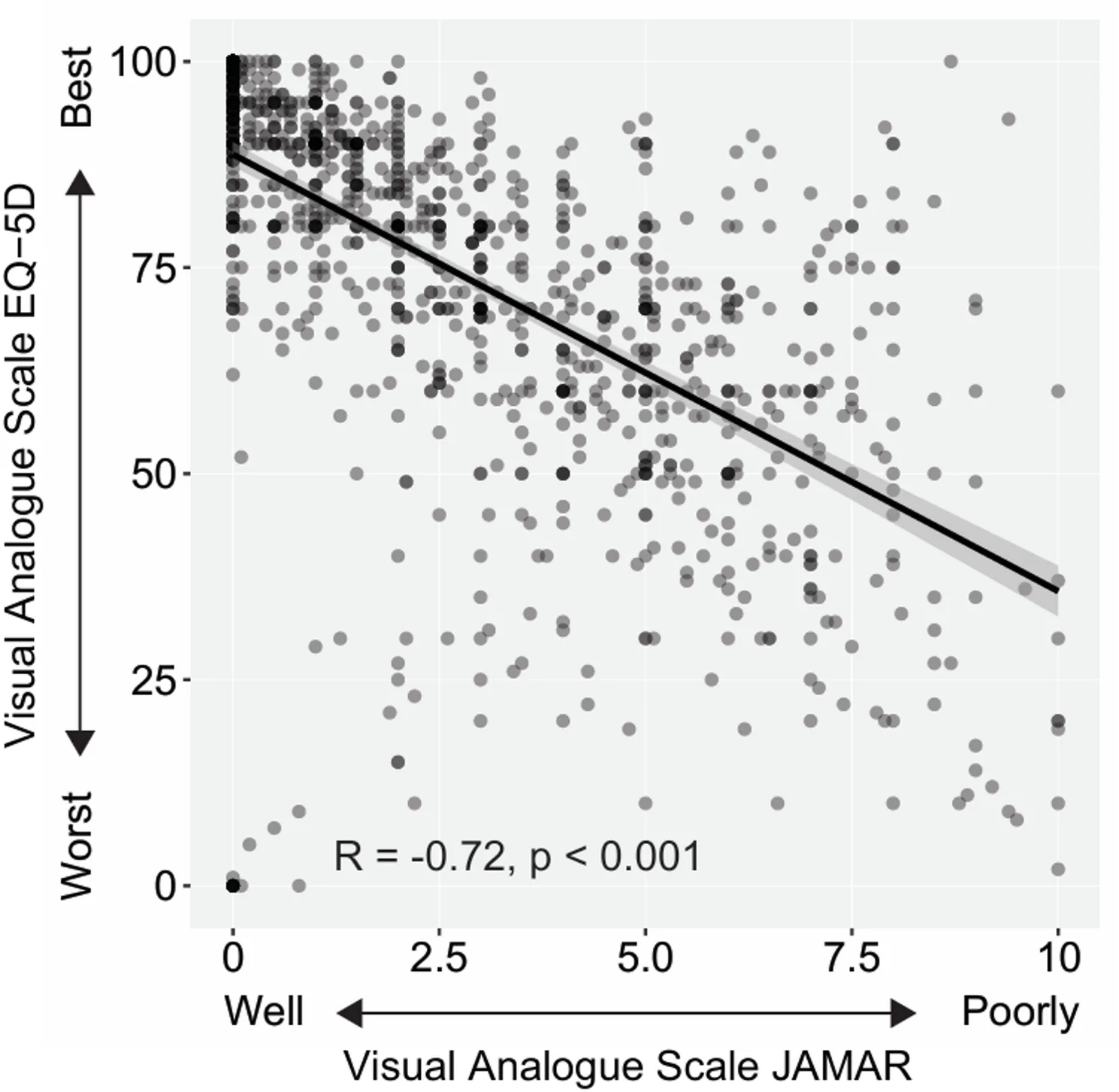
Spearman rank correlation between the global visual analogue scales of the generic (EQ-5D-Y-5L) and disease-specific (JAMAR) instruments in JIA. Spearman’s rank correlation was used to test the relationship between the EQ-5D-Y-5L question “We would like to know how good or bad your health is today” and the JAMAR question “Considering all the ways the illness affects you, please evaluate how you feel at the moment”. The EQ-5D-Y-5L question was captured using a Visual Analogue Scale from 0 (worst health you can imagine) to 100 (best health you can imagine), and the JAMAR question using a Visual Analogue Scale ranging from 0 (very well) to 10 (very poorly). A fitted trend line with its 95% confidence interval is shown for visualization. Statistically significant at P < 0.05. Abbreviations: EQ-5D-Y-5L; EuroQol 5-Dimension Youth 5-Level. JAMAR; Juvenile Arthritis Multidimensional Assessment Report. JIA; Juvenile Idiopathic Arthritis. VAS; Visual Analogue Scale

The comparison of global assessments revealed a high correlation between the EQ-5D-Y-5L VAS and the JAMAR VAS (Spearman’s correlation: *R* = -0.72, *p* < 0.001), as shown in Fig. 1.

## Discussion

This study explored the relationship between the responses to the generic EQ-5D-Y-5L instrument and the disease-specific JAMAR questionnaire of children with juvenile idiopathic arthritis and their parents. Both instruments capture distinct and overlapping aspects of health-related quality of life. The overall concordance was 84%. Domain-specific analysis revealed the highest concordance between the two questionnaires within the self-care domain (87%). Conversely, the mobility and usual activities domains showed the lowest concordance (82%) between the questionnaires when identifying problems or no problems. The concordance was reduced in all domains when children were experiencing active arthritis, except for the pain/discomfort domain. These results suggest that the responses between the generic and disease-specific HRQoL instruments are not fully comparable and that some discordance is present between the responses of the instruments.

The observed discordance in responses might be explained by differences between the EQ-5D-Y-5L and JAMAR questionnaires, including variations in response format and recall period. First, variation in questionnaire response formats may explain the discordance between responses, as the EQ-5D-Y-5L measures symptom severity, whereas the JAMAR assesses frequency. Previous research indicated that frequency and severity measures might capture distinct aspects, stating that frequency scales tend to be more sensitive in differentiating milder pain/discomfort symptoms, whereas severity scales might be more effective at distinguishing between higher pain/discomfort intensity levels [19].

Secondly, an important difference between the two questionnaires is their recall period, which may explain a substantial proportion of the discordance between the EQ-5D-Y-5L and the JAMAR responses. JIA is a fickle disease with a lot of variability in pain intensity, which can impact the HRQoL from day to day [20]. Consequently, the EQ-5D-Y-5L, which assesses health status ‘today’, may indicate no problems, whereas the JAMAR does capture problems experienced over the previous four weeks. Previous studies already reported that the use of longer recall periods results in identifying more problems [21, 22]. This aligns with our findings as well, showing that overall the JAMAR (recall period of ‘four weeks’) noted more problems than the EQ-5D-Y-5L (recall period of ‘today’). Altogether, it is important to keep the differences in response format and recall period in mind when interpreting the results.

Next to differences in response format and recall period, the respondent type could affect the level of concordance between questionnaires. Previous studies have demonstrated discrepancies between patient and parent questionnaire responses [23, 24]. Our subanalysis, restricted to patient-completed and parent-completed questionnaires, showed comparable levels of concordance between these groups. Further research into the impact of respondent type (parent versus patient) and patient age groups on concordance would be of interest, as these factors could represent potential sources of heterogeneity in concordance estimates.

## Conclusion

Responses to corresponding domains of the disease-specific JAMAR questionnaire and the generic EQ-5D-Y-5L questionnaire revealed considerable concordance. Patients with active arthritis showed lower concordance in all domains except pain/discomfort compared to patients with inactive arthritis. Overall, the disease-specific JAMAR questionnaire identified more problems than the generic EQ-5D-Y-5L in patients with JIA. Healthcare providers, researchers, and policymakers should carefully consider the differences among questionnaires when selecting assessment tools for clinical or health-economy purposes.

## Appendix: members of the UCAN CAN-DU and UCAN CURE consortia

Members of the UCAN CAN-DU and UCAN CURE consortia include: Adam Huber, Bianca Lang, Chelsea DeCoste, Elizabeth Stringer, and Suzanne Ramsey (IWK Health Centre); Alan Rosenberg, Kate Neufeld, Mehul Jariwala, and Tristan Kerr (University of Saskatchewan); Alexander Mosoiu, Alisa Rachlis, Amy Xu, Arthur Cheng, Brenleigh Jebb, Brian Feldman, Bruno Pereira, Deborah Levy, Dilan Dissanayake, Elizaveta Limenis, Evelyn Rozenblyum, Harper Cheng, Jennifer Ji Young Lee, Lynn Spiegel, Rayfel Schneider, Ronald Laxer, Ruud Verstegen, Shirley Tse, and Trang Duong (The Hospital for Sick Children); Andrea Human, David Cabral, Herman Tam, Jaime Guzman, Kim Morishita, Kristin Houghton, Lori Tucker, Mercedes Chan, Ross Petty, and Tommy Gerschman (British Columbia Children’s Hospital); Annet van Royen-Kerkhof, Berent Prakken, Erika Van Nieuwenhove, Marc Jansen, and Nico Wulffraat (Wilhelmina Children’s Hospital, University Medical Center Utrecht); Ciarán Duffy, Nadia Luca, Roman Jurencak, and Tala El Tal (Children’s Hospital of Eastern Ontario); Claire LeBlanc, Gaëlle Chédeville, Piya Lahiry, Rosie Scuccimarri, and Sarah Campillo (Montreal Children’s Hospital); Clare Hutchinson (North York General Hospital); Daniah Basodan, Dax G. Rumsey, Hon Yan Ng, Jeanine McColl, Lillian Lim, and Tara McGrath (Stollery Children’s Hospital); Danielle Brinkman and Petra Hissink Muller (Willem-Alexander Children’s Hospital, Leiden University Medical Center); Elizabeth Legger and Wineke Armbrust (Beatrix Children’s Hospital, University Medical Center Groningen); Ellen Schatorje and Esther Hoppenreijs (Amalia Children’s Hospital, Radboudumc and Sint Maartenskinderkliniek); Elodie Boudes, Gillian Currie, Heinrike Schmeling, Muhammed Dhalla, Nicole Johnson, Paivi Miettunen, Ravneet Sran, and Rebeka Stevenson (University of Calgary); Erkan Demirkaya, Jonathan Park, and Roberta Berard (Children’s Hospital, London Health Sciences Centre); Dieneke Schonenberg-Meinema, Giske Biesbroek, Mariken Gruppen and J. Merlijn van den Berg (Emma Children’s Hospital, Amsterdam University Medical Centers); Gordon Soon (North York General Hospital/Health Sciences North); Joseph Cafazzo (Centre for Global eHealth Innovation, University Health Network, University of Toronto); Liane Heale, Michelle Batthish, and Tania Cellucci (McMaster Children’s Hospital); Lily Lim (Children’s Hospital Health Centre Winnipeg); Maarten IJzerman (Erasmus University and University of Melbourne); Marinka Twilt (University of Calgary and Alberta Children’s Hospital); Marleen Verkaaik, Philomine van Pelt, and Sylvia Kamphuis (Sophia Children’s Hospital, Erasmus University Medical Center); Michelle Kip (University of Twente); Nickolas Blanchette (Trillium Health Partners); Paul Dancey (Janeway Children’s/Memorial University); and Regina de Geus (University Medical Center Utrecht).

## Supplementary Information

Below is the link to the electronic supplementary material.

Supplementary Material 1

## Data Availability

The datasets used and/or analysed during the current study are available from the corresponding author on reasonable request.

## Abbreviations

AJC: Active Joint Count
BI: Bootstrap confidence Interval
cJADAS10: clinical Juvenile Arthritis Disease Activity Scores 10 joints
EQ-5D-Y-5L: EuroQol Five-Dimension youth version with five response levels
HRQoL: Health-Related Quality of Life
ILAR: International League of Associations for Rheumatology
IQR: Interquartile Range
JAMAR: Juvenile Arthritis Multidimensional Assessment Report
JIA: Juvenile Idiopathic Arthritis
PhGA: Physician Global Assessment
PPGA: Patient/Parent Global Assessment
VAS: Visual Analogue Scale
