# Supplementary material for: Health-related quality of life in childhood arthritis: the impact of generic versus disease-specific instruments
Source: Pediatr Rheumatol Online J. 2026 Sep 12;24:79. doi: 10.1186/s12969-026-01274-9 (PMC13688762; doi:10.1186/s12969-026-01274-9)
Supplement: Supplementary file 1 [file 12969_2026_1274_MOESM1_ESM.pdf]

Supplementary Table 1: Overview of the EQ-5D-Y-5L dimensions and the corresponding JAMAR domains

EQ-5D-Y-5L dimension	JAMAR domain
Anxiety/depression	In the past four weeks, have you felt sad or depressed?
Mobility (walking)	In the past four weeks, have you had any difficulty taking a 15 minute walk or walking up a flight of stairs?
Pain/discomfort	In the past four weeks, have you had any pain?
Self-care (washing/dressing)	In the past four weeks, have you had any difficulty taking care of yourself, for example eating, getting dressed, or washing?
Usual activities (work/study/housework/family or leisure activities)	In the past four weeks, have you had any difficulty doing at-school activities or playing with friend?

Legend: Abbreviations: EQ-5D-Y-5L; EuroQol 5-Dimension Youth 5-Level. JAMAR; Juvenile Arthritis Multidimensional Assessment Report.

Supplementary Table 2: Domain-specific concordance in responses comparing generic (EQ-5D-Y-5L) and disease-specific (JAMAR) instruments by disease status based on the physician global assessment

			Number (%) of patient/parents reporting no problems versus problems on the JAMAR and EQ-5D-Y-5L				Concordance (BI)		Cohen's kappa (BI)	
			JAMAR				PhGA < 1	PhGA ≥ 1	PhGA < 1	PhGA ≥ 1
			PhGA < 1		PhGA ≥ 1					
Domain			No problems	Problems	No problems	Problems				
**Anxiety/ depression**	**EQ-5D-Y-5L**	No problems	214 (59)	32 (9)	160 (33)	36 (7)	85%	83%	0.66	0.66
		Problems	22 (6)	93 (26)	45 (9)	245 (50)	(81%-89%	(80%-87%)	(0.57-0.74)	(0.58-0.72)
**Mobility**		No problems	244 (67)	41 (11)	123 (25)	66 (14)	84%	80%	0.57	0.56
		Problems	17 (5)	61 (17)	32 (7)	265 (55)	(80%-88%)	(76%-84%)	(0.47-0.67)	(0.48-0.64)
**Pain/ discomfort**		No problems	161 (44)	48 (13)	35 (7)	41 (8)	79%	88%	0.57	0.47
		Problems	29 (8)	126 (35)	20 (4)	397 (81)	(74%-83%)	(85%-90%)	(0.49-0.66)	(0.34-0.57)
**Self-Care**		No problems	300 (85)	25 (7)	243 (54)	59 (13)	92%	82%	0.57	0.60
		Problems	5 (1)	24 (7)	24 (5)	123 (27)	(88%-95%)	(78%-85%)	(0.41-0.71)	(0.53-0.68)
**Usual activities**		No problems	230 (66)	31 (9)	107 (24)	32 (7)	83%	82%	0.57	0.59
		Problems	27 (8)	62 (18)	50 (11)	256 (58)	(79%-87%)	(78%-85%)	(0.47-0.67)	(0.51-0.67)

Legend: The numbers of patients/parents reporting no problems versus problems on the JAMAR and EQ-5D-Y-5L, by disease state based on the PhGA, are presented as n (% of total within each domain). "No problems" were defined as responses of *never* on the JAMAR and *no problems* on the EQ-5D-Y-5L. "Problems" included all other response categories (JAMAR: *sometimes, often, every day*; EQ-5D-Y-5L: *a little bit, some, a lot, extreme*). Observed concordance was calculated based on dichotomized JAMAR and EQ-5D-Y-5L responses and presented as a percentage. Cohen's kappa was calculated to assess agreement while accounting for agreement expected by chance. Cluster-level bootstrap resampling (2000 replicates) was performed at the patient level, and bootstrap confidence intervals were reported to account for non-independence of observations within patients. Abbreviations: BI; Bootstrap confidence Interval. EQ-5D-Y-5L; EuroQol 5-Dimension Youth 5-Level. JAMAR; Juvenile Arthritis Multidimensional Assessment Report. PhGA; Physician Global Assessment.

Supplementary Figure 1: Number of paired generic (EQ-5D-Y-5L) and disease-specific (JAMAR) questionnaires completed by JIA patients or parents

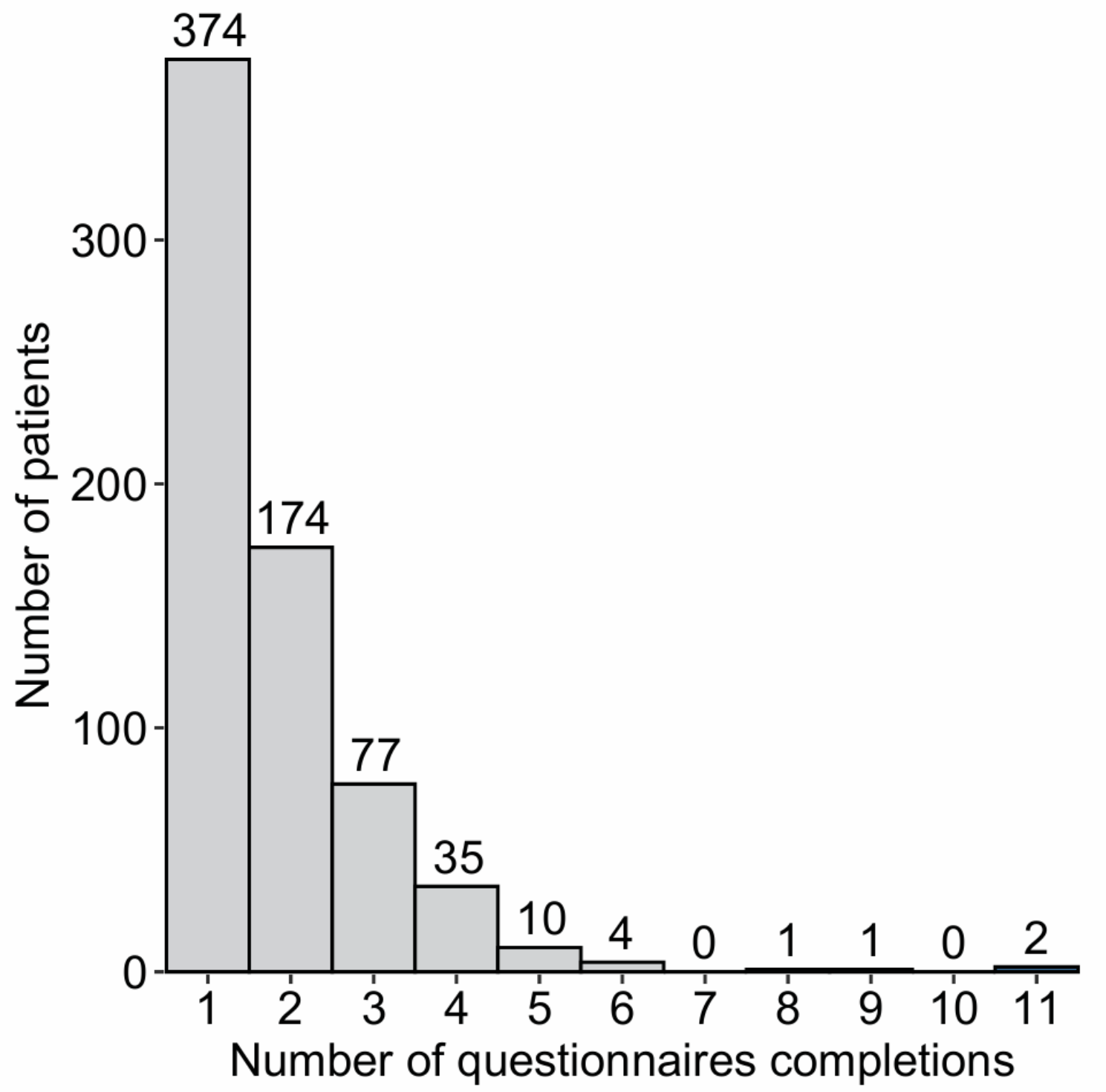

Legend: A total of 1206 paired generic (EQ-5D-Y-5L) and disease-specific (JAMAR) questionnaires were completed by 678 JIA patients/parents. Abbreviations: EQ-5D-Y-5L; EuroQol 5-Dimension Youth 5-Level. JAMAR; Juvenile Arthritis Multidimensional Assessment Report. JIA; Juvenile Idiopathic Arthritis.
